# Lack of the Actin Capping Protein, Eps8, Affects NMDA-Type Glutamate Receptor Function and Composition

**DOI:** 10.3389/fnmol.2018.00313

**Published:** 2018-09-05

**Authors:** Raffaella Morini, Silvia Ferrara, Fabio Perrucci, Stefania Zambetti, Silvia Pelucchi, Elena Marcello, Fabrizio Gardoni, Flavia Antonucci, Michela Matteoli, Elisabetta Menna

**Affiliations:** ^1^Laboratory of Pharmacology and Brain Pathology, Neurocenter IRCCS Humanitas, Milan, Italy; ^2^Dipartimento di Biotecnologie Mediche e Medicina Traslazionale, Università di Milano, Milan, Italy; ^3^Department of Biomedical Sciences, Humanitas University, Milan, Italy; ^4^Dipartimento di Scienze Farmacologiche e Biomolecolari (DiSFeB), Università di Milano, Milan, Italy; ^5^NEUROFARBA, Dipartimento di Neuroscienze, Psicologia, Area del Farmaco e Salute del Bambino, Università degli Studi di Firenze, Florence, Italy; ^6^CNR-Istituto di Neuroscienze (IN), Milan, Italy

**Keywords:** EPS8, synapse, NMDA receptor, actin cytoskeleton, GluN2A, GluN2B

## Abstract

Actin-based remodeling underlines spine morphogenesis and plasticity and is crucially involved in the processes that constantly reshape the circuitry of the adult brain in response to external stimuli, leading to learning and memory formation and supporting cognitive functions. Hence spine morphology and synaptic strength are tightly linked and indeed abnormalities in spine number and morphology have been described in a number of neurological disorders such as autism spectrum disorders (ASDs), schizophrenia and intellectual disabilities. We have recently demonstrated that the actin regulating protein, Epidermal growth factor receptor pathway substrate 8 (Eps8), is essential for spine growth and long term potentiation. Indeed, mice lacking Eps8 display immature filopodia-like spines, which are unable to undergo potentiation, and are impaired in cognitive functions. Furthermore, reduced levels of Eps8 have been found in the brain of a cohort of patients affected by ASD compared to controls. Here we investigated whether the lack of Eps8, which is also part of the N-methyl-d-aspartate (NMDA) receptor complex, affects the functional maturation of the postsynaptic compartment. Our results demonstrate that Eps8 knock out mice (Eps8 KO) neurons display altered synaptic expression and subunit composition of NMDA receptors (i.e., increased GluN2B-, decreased GluN2A-containing receptors) and impaired GluN2B to GluN2A subunit shift. Indeed Eps8 KO neurons display increased content of GluN2B containing NMDA receptors both at the synaptic and extrasynaptic level. Furthermore, Eps8 KO neurons display an increased content of extra-synaptic GluN2B-containing receptors, suggesting that also the synaptic targeting of NMDA receptors is affected by the lack of Eps8. These data demonstrate that, besides regulation of spine morphogenesis, Eps8 also regulates the synaptic balance of NMDA receptors subunits GluN2A and GluN2B.

## Introduction

Filamentous actin (F-actin) represents the major cytoskeletal component of dendritic spines (Fifková and Delay, 1982; Cohen et al., 1985), controlling spine shape, size and number through local, rapid changes in its dynamics (Matus et al., 1981; Fischer et al., 1998). Actin is highly enriched at the postsynaptic density (PSD) where, through the interaction with a cohort of scaffolding proteins (Qualmann et al., 2004), it anchors receptors, thus acting as a key determinant of synaptic strength (Sheng and Hoogenraad, 2007). Accordingly, F-actin depolymerization disperses α-amino-3-hydroxy-5-methyl-4-isoxazole propionic acid (AMPA) and N-methyl-d-aspartate (NMDA) receptors at excitatory synapses and reduces the clusters of gephyrin, a glycine receptor scaffolding protein, at inhibitory synapses (Allison et al., 1998; Hanley, 2014). Actin cytoskeleton also regulates postsynaptic receptor mobility in and out of synapses. As an example, in the case of glycine receptors, disruption of F-actin enhances the rate of exchange of synaptic and extrasynaptic receptors, while decreasing receptor “dwell” time at synapses and increasing the diffusion of receptor subsets within synapses (Charrier et al., 2006; Dumoulin et al., 2009). Therefore, rather than being a “molecular glue,” actin appears to crucially contribute to the differential organization of distinct pools of receptors promoting the potential sub-cluster associations within the postsynaptic specialization. A similar function for actin might extend to excitatory synapses, that harbor AMPA-type and NMDA-type glutamate receptors, which are differentially sensitive to actin depolymerization (reviewed in Cingolani and Goda, 2008).

NMDA receptors (thereafter referred as NMDARs) are hetero-tetramers comprising numerous combinations of GluN1 and GluN2A-D subunits, which serve for the binding to co-agonist or to glutamate respectively (see for a review Paoletti et al., 2013). Many of the unique properties of NMDARs have been invariably attributed to the diversity of the individual subunits and their combinations to form the receptor (Wyllie et al., 2013). NMDAR composition is developmentally regulated: GluN2B subunits are highly expressed during early development and reach a peak around the second postnatal week, whereas GluN2A subunit levels increase only after birth, exceeding GluN2B subunits by adulthood (Monyer et al., 1994; Barth and Malenka, 2001). The progressive increase of GluN2A at developing synapses is essential for synaptic maturation, and mature neuronal network establishment (Yashiro and Philpot, 2008). NMDARs, both GluN1 and GluN2 subunits, are immediately associated with the cytoskeleton via protein–protein interactions with F-actin (Wyszynski et al., 1997; Wechsler and Teichberg, 1998). The integrity of the cytoskeleton influences the activity of NMDARs (Rosenmund and Westbrook, 1993) and, in turn, activation of NMDARs can trigger depolymerization of actin (Sattler et al., 2000). Actin dynamics are controlled by ensembles of actin-binding proteins (Konietzny et al., 2017). These proteins play different functional roles in regulating actin dynamics, including binding and/or sequestering of actin monomers, nucleation of actin filaments, capping or anti-capping of barbed ends, and severing, bundling and anchoring of F-actin (Nicholson-Dykstra et al., 2005; Pak et al., 2008; Shekhar et al., 2016). In particular actin capping proteins regulate actin polymerization and dynamics by binding the barbed end of an actin filament and blocking addition and loss of actin subunits (Edwards et al., 2014). Hence actin capping proteins play a key role in maintaining the integrity of actin cytoskeleton (Hotulainen and Hoogenraad, 2010).

Epidermal growth factor receptor pathway substrate 8 (Eps8) is a multi-functional actin-binding protein which participates, via its SH3 domain, in the formation of distinct macromolecular complexes that transduce signals from Ras to Rac and control actin capping and bundling activities (Scita et al., 1999; Disanza et al., 2004, 2006). During neuronal development, Eps8, by inhibiting actin elongation, down-regulates axonal filopodia formation in neurons with Eps8 phosphorylation leading to inhibition of its actin-capping function and stimulation of filopodia extension (Menna et al., 2009). Consistently, absence of Eps8 has been found to alter the growth of vestibular hair cells stereocilia (Tavazzani et al., 2016).

In mature primary hippocampal neurons, Eps8 is recruited to the spine head during long-term potentiation promoting spine head enlargement, which typically occurs during potentiation processes. Indeed, inhibition of Eps8 actin-capping activity impairs spine enlargement, leading to an excessive formation filopodia-like spines which are unable to undergo plasticity (Menna et al., 2013). Accordingly, mice lacking Eps8 (Eps8 knock out mice, Eps8 KO) display increased density of immature spines, which is correlated with a learning and memory impairment of Eps8 deficient mice (Menna et al., 2013). Furthermore, Eps8 levels were found to be reduced in the brains of a cohort of patients affected by autism spectrum disorders (ASDs; Menna et al., 2013). Eps8 has been found to be expressed in cerebellar glomeruli, with a pattern of expression typically postsynaptic and coinciding with that of F-actin. Here, Eps8 was found to interact with the NMDAR subunits, GluN2A, GluN2C and GluN1, but not with GluA1 subunit of AMPA-type receptors, indicating Eps8 as part of the NMDAR complex (Offenhauser et al., 2006). Eps8 controls granule cell NMDA postsynaptic currents, with Eps8 KO cerebellar granule neurons displaying increased current amplitude and slower decay kinetics (Offenhauser et al., 2006).

Based on these experimental results we investigated whether Eps8 may control the expression and function of NMDARs or specific NMDAR subunits. Using Eps8 KO neurons and brain extracts, we analyzed the GluN2A/2B subunit ratio in NMDARs and we demonstrate that lack of Eps8 affects NMDAR expression, synaptic localization and function by increasing the amount of GluN2B subunits at the synaptic and extra-synaptic level while amount of synaptic GluN2A-containing receptors is reduced.

## Materials and Methods

### Cell Cultures

All the experimental procedures followed the guidelines established by the Italian Council on Animal Care and were approved by the Italian Government Decree No. 27/2010 (see Supplementary Information) and the Italian Legislation (L.D. no. 26/2014). All efforts were made to minimize the number of animals used and their sufferings. All animals were housed with 12/12-h light/dark cycle with food and water available *ad libitum*. Embryonic (E18) primary cultures of mouse hippocampal neurons were established from Eps8 KO^1^ and wild type (WT) mice obtained from breeding settled up in our animal facility as described by Menna et al. (2013). Briefly, hippocampi were dissociated by treatment with trypsin (0.125% for 15 min at 371°C), followed by trituration with a polished Pasteur pipette. The dissociated cells were plated onto glass coverslips coated with poly-L-lysine at density of 400 cells/mm^2^. For low density neuronal cultures used in paired electrophysiological experiments, dissociated cells were plated onto glass coverslips coated with poly-L-lysine at density of 150–200 cells/mm^2^. The cells were maintained in Neurobasal (Invitrogen) with B27 supplement and antibiotics, 2 mM glutamine and 12.5 mM glutamate (neuronal medium).

### Subcellular Fractionation

Western blot analysis was performed in hippocampal tissue of Eps8 KO and WT adult (5 months of age) littermates mice. To obtain a preparation that contains selectively proteins of the excitatory PSD, subcellular fractionation was performed as reported previously with minor modifications (Gardoni et al., 2001b). Hippocampi were homogenized in 0.32 M ice-cold sucrose containing the following (in mM): 1 HEPES, 1 MgCl_2_, 1 EDTA, 1 NaHCO_3_ and 0.1 PMSF at pH 7.4, in the presence of a complete set of proteases inhibitors (Complete; Roche Diagnostics) and phosSTOP Phosphatase Inhibitor (Roche Diagnostics). The homogenized tissue was centrifuged at 13,000× *g* for 10 min (P2 fraction). The pellet was resuspended in buffer containing 75 mM KCl and 1% Triton X-100 and centrifuged at 100,000× *g* for 1 h. The supernatant was stored and referred as Triton X-100-soluble fraction (TSF). The final pellet (triton insoluble fractions, TIF) was homogenized in a glass–glass potter in 20 mM HEPES. TIF was used instead of the classical PSD because the amount of the starting material was very limited. The protein composition of this preparation was, however, carefully tested for the absence of presynaptic markers (i.e., synaptophysin; Gardoni et al., 2009). Similar protein yield was obtained in TIF purified from hippocampal tissue of all experimental groups.

### Western Blot (WB)

WB analysis was performed in homogenate and TIF fractions. Protein samples were separated onto an acrylamide/bisacrylamide gel at the appropriate concentration and transferred to a nitrocellulose membrane. Nitrocellulose articles were blocked with 10% albumin in tris-buffered saline (TBS) and then incubated overnight at 4° with the primary antibodies. After extensive rinsing in TBS/0.1% Tween 20, the nitrocellulose articles were then incubated with horseradish peroxidase-conjugated secondary antibodies (goat anti-rabbit, for polyclonal antibodies, diluted 1:10,000 (Pierce); goat anti-mouse, for monoclonal antibodies, diluted 1:10,000 (Pierce). Membrane development was performed with the reagent Clarity Western ECL Substrate (Bio-Rad) or LiteAblot TURBO (Euroclone) and labeling was visualized by Chemidoc Imaging System and ImageLab software (Bio-Rad). For quantification, each protein was normalized against the corresponding tubulin band. The following unconjugated primary antibodies were used: GluN2A (diluted 1:1,000; Sigma-Aldrich M264), GluN2B (diluted 1:1,000; Neuromab 75–101), PSD-95 (diluted 1:2,000; Neuromab 75–028), tubulin (diluted 1:10,000; Sigma-Aldrich T9026), Phospho-p44/42 MAPK (Erk1/2; Thr202/Tyr204; diluted 1:2,000; Cell signaling 9101), p44/42 MAPK (Erk1/2; diluted 1:2,000; Cell signaling 9102), total GluN2B (Mellone et al., 2015) p1472 (diluted 1:1,000; Calbiochem 454583).

### Surface Staining for GluN2B

Living neurons were incubated for 10 min with rabbit antibodies directed against extracellular epitopes of GluN2B (diluted 1:100, Alomone Labs AGC-003), washed and fixed with 4% paraformaldehyde and 4% sucrose as described (Joshi et al., 2017). The guinea pig anti-Bassoon (diluted 1:500, Synaptic System 141004) was used to double stain the cultures. Secondary antibodies were conjugated with Alexa-488 and Alexa-555 (Alexa-Invitrogen). Images were acquired using a Zeiss LSM800 confocal microscope equipped with a Plan-Apochromat 63×/1.40 oil objective. Acquisition parameters (i.e., laser power, gain and offset) were kept constant among different experimental settings. Surface synaptic GluN2B staining was quantified as follows. GluN2B-, bassoon-positive puncta and GluN2B puncta colocalizing with bassoon (both number and size) were measured using ImageJ 1.46r software in GluN2B and bassoon confocal images after setting a fixed threshold using the “analyze particle” and “image processing” functions. The “*synaptic surface GluN2B*” is calculated by the ratio of the “*area of surface GluN2B colocalizing with bsn/area of surface GluN2B*.” Approximately 10–15 fields were acquired per independent experiments. All data are results of at least three independent experiments.

For the staining of F actin, vehicle- and Latrunculin B (LatB)-treated cultures were fixed with 4% paraformaldehyde and 4% sucrose as described (Bedogni et al., 2016). AlexaFluor555 conjugated–phalloidin (diluted 1:200, Molecular Probes A34055) and the mouse monoclonal PSD-95 (diluted 1:400, Neuromab 75–028) were used.

### Cell Culture Electrophysiology

Whole cell voltage-clamp recordings were performed on WT and transgenic E18 hippocampal neurons maintained in culture for 13–15 DIV. During recordings cells were bathed in a standard external solution containing (in mM): 125 NaCl, 5 KCl, 1.2 MgSO_4_, 1.2 KH_2_PO_4_, 2 CaCl_2_, 6 glucose and 25 HEPES-NaOH, pH 7.4. Recording pipettes were fabricated from borosilicate glass capillary using an horizontal puller (Sutter Instruments) inducing tip resistances of 3–5 MΩ and filled with a standard intracellular solution containing (in mM): 130 Cs-gluconate, 8 CsCl, 2 NaCl, 4 EGTA, 10 HEPES-NaOH, 2 MgCl_2_, 4 MgATP and 0.3 Tris-GTP.

For miniature AMPA-NMDA EPSC recordings, cells were bathed in a free Mg^2+^ solution containing (in mM) 125 NaCl, 5 KCl, 1.2 KH_2_PO_4_, 2 CaCl_2_, 6 glucose and 25 HEPES-NaOH, tetrodotoxin (TTX) 0.001, Strychnine 0.001 and bicuculline methiodide 0.02, glycine 0.01 (pH 7.4; Tocris). To record the pure NMDA currents 6-cyano-7-dinitroquinoxaline-2,3-dione (CNQX) 20 μM (Tocris) were added to standard extracellular solution to block the AMPA component of the current. The patch pipette electrode contained the following (in mM): 130 CsGluconate, 8 CsCl, 2 NaCl, 10 HEPES, 4 EGTA, 4 MgATP and 0.3 Tris-GTP.

AMPA and NMDA evoked currents were recorded in isolated pairs of neurons in low-density cultures. Neurons were bathed in a standard external solution containing (in mM): 125 NaCl, 5 KCl, 1.2 MgSO_4_, 1.2 KH_2_PO_4_, 2 CaCl_2_, 6 glucose and 25 HEPES-NaOH, pH 7.4. Neurons were held at −70 mV or at +40 and eEPSC evoked by a 100-mV depolarization pulse in the presynaptic cell lasting 1 ms. The AMPA/NMDA ratio was calculated by estimating the respective AMPA and NMDAR current on the traces at +40 mV based on their different time courses (NMDA component was calculated 40 ms after stimulus artifact).

For whole-cell total currents recording were bathed in a free Mg^2+^ solution containing (in mM) 125 NaCl, 5 KCl, 1.2 KH_2_PO_4_, 2 CaCl_2_, 6 glucose and 25 HEPES-NaOH, TTX 0.001, Strychnine 0.001 and bicuculline methiodide 0.02, glycine 0.01 (pH 7.4). NMDA whole-cell total currents were elicited by fast local application of saturating NMDA concentrations, which activate both synaptic and extrasynaptic receptors. NMDA (200 μM) will be applied with or without inhibitors (ifenprodil 3 μM or Zinc 10 nM in zinc-free tricine solution to block GluN2B and GluN2A respectively; Paoletti et al., 1997).

To measure rectification, spontaneous AMPA-mediated currents were measured at −60 and at +50 mV, under conditions in which spontaneous AMPA currents could be isolated. For these experiments the ACSF contained 0.01 μM TTX (to raise spike threshold), APV (100 μM) and MK801 (50 μM) to block NMDA currents, and bicuculline (20 μM) to block inhibitory potentials. The internal solution contained spermine (120 μM) was Cs-based to facilitate voltage-clamping at +50 mV. To compute the rectification index the average peak amplitude at +50 was divided by the average peak amplitude at −60; the smaller this value, the more rectification. To induce synaptic scaling, neurons were treated with 1 μM TTX for 48 h and then mEPSC currents were recorded.

For the analysis of the tonic, inward, noisy current recordings were carried out in [0 Mg^2+^]_e_, 20 μM CNQX, bicuculline (50 μM) and TTX (1 μM), before and after blocking NMDAR function with APV (50 μM) or ifenprodil (3 μM). Two alternative approaches were used to quantify tonic NMDAR current. First, it was assessed as the shift in holding current resulting from NMDA blocker (AP5) application, second, AP-5-associated change in a baseline noise. Quantification of background noise was obtained plotting all values of recordings traces and comparing the fluctuation of values distribution.

Recordings were performed at room temperature in voltage clamp mode using a Multiclamp 700B amplifier (Molecular Devices) and pClamp-10 software (Axon Instruments). Series resistance ranged from 10 MΩ to 20 MΩ and was monitored for consistency during recordings. Cells in culture with leak currents >100 pA were excluded from the analysis. Signals were amplified, sampled at 10 kHz, filtered to 2 or 3 KHz and analyzed using pClamp 10 data acquisition and analysis program.

### Statistical Analysis

Statistical analysis was performed using Prism6 (GraphPad), data are presented as mean ± SEM from the indicated number of experiments. After testing whether data were normally distributed or not, the appropriate statistical test, followed by specific multiple comparison *post hoc* tests, has been used as indicated in figure legends. Kolmogorov–Smirnov test was used to determine significance in cumulative distributions of mEPSC amplitudes. Differences were considered to be significant if *P* < 0.05 and are indicated by one asterisk; those at *P* < 0.01 are indicated by double asterisks; those at *P* < 0.001 are indicated by triple asterisks, those at *P* < 0.0001 are indicated by four asterisks.

## Results

### Eps8 KO Neurons Display Reduced NMDA-Mediated Synaptic Activity

**Figure 1:**
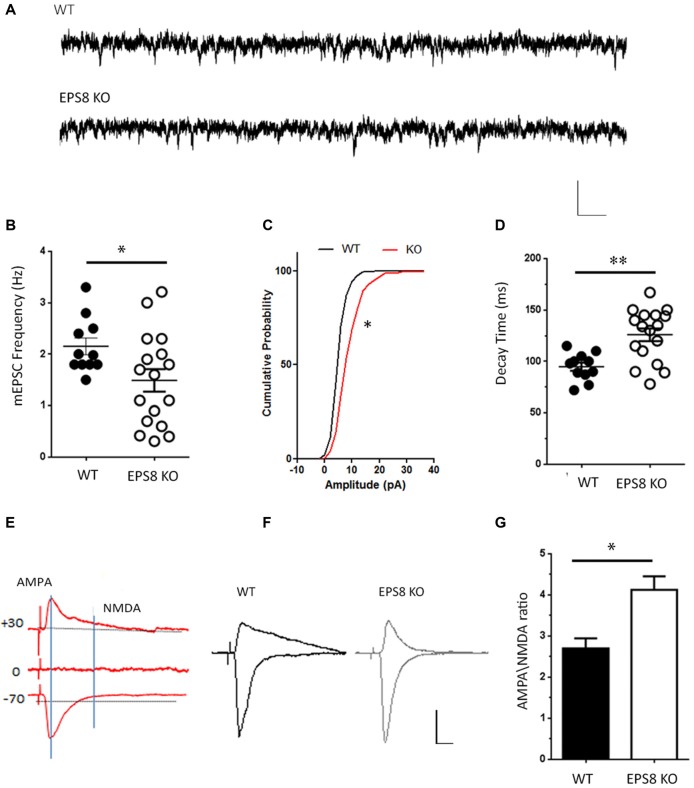
Epidermal growth factor receptor pathway substrate 8 (EPS8) deficiency reduces N-methyl-d-aspartate (NMDA)-mediated synaptic activity. **(A)** Representative traces of NMDA receptor-mediated mEPSCs (NMDA-mEPSC) recorded from mature wild type (WT) and Eps8 knock out mice (Eps8 KO) primary hippocampal neurons. Scale bars 10 pA, 50 ms. **(B–D)** Quantitation of mEPSC frequency, cumulative probability plot of mEPSC amplitudes and decay time showing a reduced frequency (Unpaired *t*-test, *P = 0.0374) and an increased amplitudes (Kolmogorov-Smirnov test, * = 0.0348, *D* = 0.4500) and decay time (Unpaired *t*-test, **P = 0.001) in Eps8 KO with respect to wt neurons. **(E)** Graphical scheme to show the difference in time course of α-amino-3-hydroxy-5-methyl-4-isoxazole propionic acid (AMPA) and NMDA currents. The AMPA component was obtained by measuring the EPSC peak amplitude at −70 mV; the NMDA component was determined by measuring the current amplitude at 100 ms after EPSC onset at +40 mV (vertical line). **(F)** Examples of evoked EPSCs composed of AMPAR and NMDAR-mediated components recorded in WT and EPS8 KO hippocampal neurons. Scale bars 20 pA, 5 s. **(G)** AMPA/NMDA ratio analysis showing a statistically significant increase of AMPA/NMDA ratio in Eps8 KO neurons (data are shown as mean ± SEM; Mann Whitney test *P = 0.0159).

To test whether the lack of Eps8 affects NMDAR function, NMDA-mediated miniature excitatory currents (mEPSCs) were recorded in mature WT and Eps8 KO primary hippocampal neurons (DIV 14–15) in Mg^2+^ free solution in the presence of TTX (1 μM) to block action potential-mediated release, the GABA-A receptor antagonist bicuculline (20 μM) and the selective antagonist of AMPA/kainate glutamate receptors CNQX (20 μM). A significantly lower frequency of NMDA-mediated mEPSCs was recorded in mutant neurons compared to WT (Hz, WT = 2.153 ± 0.162, *n* = 11 cells; EPS8 KO = 1.491 ± 0.218, *n* = 17 cells, Unpaired *t*-test *P = 0.0374 data are expressed and mean ± SEM; Figures 1A,B), suggesting a reduced NMDA synaptic component in mature Eps8 KO hippocampal cultures.

In line with a reduced NMDA synaptic component, the NMDA evoked current appeared to be lower in Eps8 KO neurons relative to controls. Indeed, temporal separation of AMPA and NMDA evoked components (obtained by maintaining neurons in 1.2 mM Mg^2+^ to block NMDA currents and record the AMPA component at –70 mV, or by stimulating neurons at +30 mV to record the NMDAR component of the EPSC) revealed a significant increase of the AMPA/NMDA ratio in Eps8 KO neurons (WT = 2.7 ± 0.2, *n* = 9; KO = 4.12 ± 0.3, *n* = 5; Mann Whitney test *P = 0.0159; Figures 1E–G). On the contrary the AMPA evoked component, obtained by measuring the EPSC peak amplitude at −70 mV (see also “Materials and Methods” section), was not changed between WT and Eps8 KO neurons (pA, WT = 37.2 ± 11, *n* = 9; KO = 35.4 ± 5.5, *n* = 6; Mann Whitney test *P* = 0.9).

Notably, Eps8 KO miniature NMDA currents displayed a larger amplitude and a slower decay time (pA: WT = 8.527 ± 0.3878; EPS8 KO = 10.7 ± 0.7462; data shown as cumulative probability, Kolmogorov-Smirnov test, * = 0.0348, *D* = 0.4500. Decay time, ms: WT = 94.8 ± 4; EPS8 KO = 125 ± 6; Unpaired *t*-test **P = 0.0010; Figures 1C,D).

Altogether these results indicate that Eps8 KO hippocampal neurons display reduced NMDAR synaptic activity which is also characterized by higher amplitude and a slower decay time, raising the possibility that mutant synapses may be endowed with different subtypes of NMDAR subunits (Cull-Candy and Leszkiewicz, 2004; Erreger et al., 2005; Santucci and Raghavachari, 2008).

### Lack of Eps8 Affects the Subunit Composition of NMDARs

Given that Eps8 KO hippocampal neurons display slower decay of NMDA current (Figure 1D), we assessed the possible occurrence of GluN2-related differences in NMDAR stoichiometry at the mutant and WT synapses. To probe the subunit composition for GluN2B and GluN2A, we tested the effect of ifenprodil, a GluN2B subunit-specific antagonist of NMDARs (Tovar and Westbrook, 1999; Cull-Candy et al., 2001), in 14–15 DIV WT and Eps8 KO primary hippocampal cultures.

**Figure 2:**
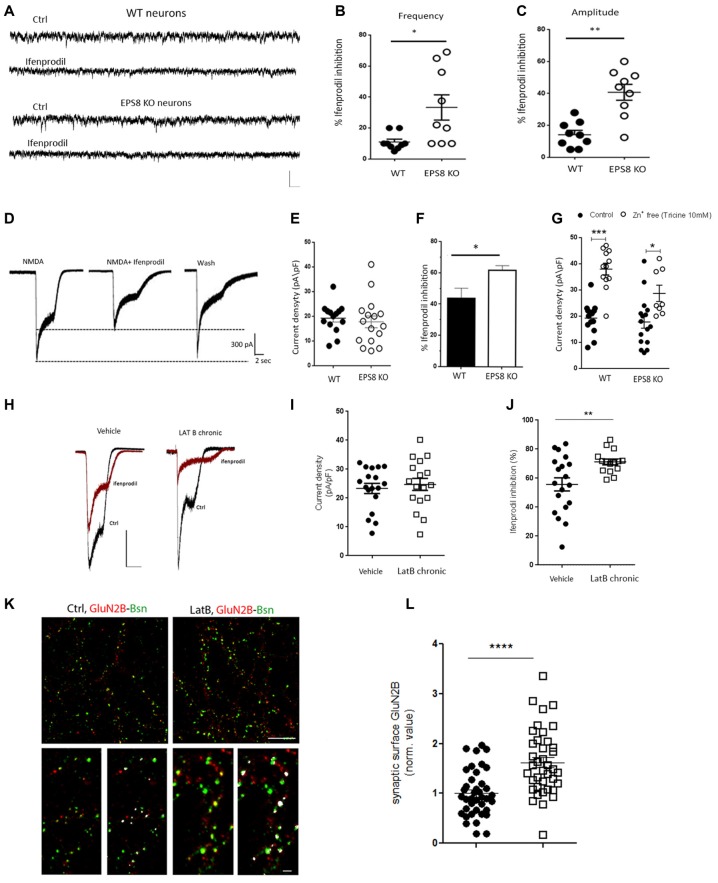
Increased GluN2B and reduced GluN2A synaptic content of NMDARs in Eps8 KO neurons. **(A)** Representative traces of NMDAR mediated mEPSCs recorded from WT and EPS8 KO hippocampal neurons in the presence or not of the GluN1/GluN2B blocker Ifenprodil (3 μM). Scale bar 10 pA, 50 ms. **(B,C)** Quantification of the inhibitory effect of ifenprodil on the NMDA-mEPSC frequency and amplitude respectively showing increased inhibition in KO neurons with respect to WT (B: Mann Whitney Test *P = 0.0270; C: Unpaired t test **P = 0.0065). **(D)** Examples of whole-cell currents elicited in WT and EPS8 KO hippocampal neurons by application of a saturating concentration of NMDA (200 μm) in the presence or absence of GluN2B-selective antagonist ifenprodil (3 μM). Scale bar 300 pA, 2 s. Quantitation of current density **(E)**, %age of ifenprodil inhibition **(F)** showing a larger amount of ifenprodil-dependent inhibition in KO neurons with respect to WT (Mann Whitney Test *P = 0.0127). **(G)** Summary distribution graph of whole-cell currents elicited in WT and EPS8 KO treated or not with tricine (10 mM) showing a reduced GluN2A-dependent increase of current density upon tricine exposure in KO neurons with respect to WT (Mann Whitney Test, ***P <0.001 and *P = 0.0220). **(H)** Representative traces of whole-cell currents elicited in Vehicle and Latrunculin B (LatB)-treated neurons by application of a saturating concentration of NMDA (200 μm) in the presence or absence of GluN2B-selective antagonist ifenprodil (3 μM).Scale bar 500 pA, 2 s Quantitation of current density **(I)**, %age of ifenprodil inhibition **(J)** showing a larger amount of ifenprodil-dependent inhibition in LatB-treated neurons with respect to vehicle treated neurons (Unpaired *t*-test **P = 0.0078). **(K)** Images of neurons live stained for GluN2B (red), fixed and counterstained against the presynaptic marker bassoon (green). Scale bar depicts 10 μm for the low magnification image and 3 μm for the higher magnification image. **(L)** The dot blot distribution graph shows quantification of the surface synaptic GluN2B signal (i.e., area of GluN2B puncta colocalizing with bassoon positive puncta/total area of GluN2B (data are mean ± SEM, *N* = 3; Mann-Whitney Rank Sum Test, ****P ≤ 0.0001).

Three micromolar ifenprodil, a concentration which does not affect GluN2A-containing receptors (Tovar and Westbrook, 1999), blocked ~40% of the synaptic NMDA-mediated mEPSCs in Eps8 KO neurons vs. ~10% in WT neurons (Hz, WT ctrl: 2.130 ± 0.15, *n* = 8 cells, WT + ifenprodil: 1.99 ± 0.18 *n* = 9 cells; EPS8 KO Ctrl: 1.432 ± 0.11 *n* = 12 cells, EPS8 KO + ifenprodil: 1.044 ± 0.127, *n* = 9 cells; pA: WT Ctrl: 8.4 ± 0.3, WT + ifenprodil: 7.675 ± 0.6; EPS8 KO Ctrl: 10.8 ± 0.78, EPS8 KO + ifenprodil: 7.0 ± 0.7 Mann Whitney Test *P = 0.0270; Unpaired *t*-test **P = 0.0065; Figures 2A–C) indicating that mature EPS8 KO primary hippocampal cultures display an altered GluN2B/2A synaptic composition. To further investigate the contribution of specific receptor subtypes to NMDA currents in WT vs. Eps8 KO neurons, we recorded the total current produced by fast local application of saturating NMDA concentrations (200 μM, 5 s), which activate both synaptic and extrasynaptic NMDARs. NMDA application produced large responses, with a comparable amplitude between WT and EPS8 KO neurons (pA/pF: WT, 19.24 ± 1.5 *n* = 14 cells; EPS8 KO, 17.76 ± 2 *n* = 16 cells; Unpaired *t*-test *P* = 0.6200; Figures 2D,E). However, mutant neurons were more sensitive to the non-competitive GluN2B blocker, as shown by the significantly larger inhibition of NMDA-mediated total current in EPS8 KO hippocampal neurons exposed to 3 μM ifenprodil with respect to WT neurons (% ifenprodil inhibition: WT, 43.71 ± 6 *n* = 8 cells; EPS8 KO, 61.8 ± 3 *n* = 8 cells, Mann Whitney Test *P = 0.0127; Figure 2F). This evidence is consistent with a higher amount of GluN2B-containing receptors in mutant neurons. Notably, exposure of WT and KO neurons to tricine (10 mM), a chelator of Zn^2+^ ions which tonically inhibits GluN2A-contaning NMDARs by binding to the Zn^2+^ binding site on the GluN2A subunits (Paoletti et al., 1997), resulted in enhanced NMDA responses at a significantly higher extent in WT relative to KO neurons (Figure 2G; Mann Whitney Test, ***P < 0.001 and *P = 0.0220). These data reveal a reduced GluN2A- and an increased GluN2B-component of NMDAR activity in Eps8 KO hippocampal neurons with respect to age-matched WT primary hippocampal cultures suggesting that the lack of Eps8 affects the process of NMDAR complex maturation in neurons.

Since Eps8 regulates actin dynamics in filopodia and spines, we aimed to assess whether the integrity of the actin cytoskeleton affects the subunit composition of NMDARs. To this aim, primary cultured hippocampal neurons were treated with LatB (300 nM from 10 DIV to 14 DIV) to depolymerize actin and disrupt its coupling to NMDARs during synapse development *in vitro* (Allison et al., 1998; Sattler et al., 1999). We then measured the total current produced by fast local application of NMDA (200 μM, 5 s), before and after bath application of ifenprodil (3 μM). Chronic treatment with LatB does not affect the current density of NMDA responses (pA/pF: vehicle, 23.34 ± 1.752 *n* = 18 cells; LatB chronic, 24.71 ± 2.09 *n* = 17 cells; Unpaired *t*-test *P* = 0.602; Figures 2H,I). However, LatB-treated cultures were more sensitive to the GluN2B blocker, ifenprodil, as shown by the increased percentage of inhibition of NMDA currents (% ifenprodil inhibition: vehicle, 55.73 ± 4.5 *n* = 19 cells; LatB chronic, 71.21 ± 1.9 *n* = 15 cells, Unpaired *t*-test **P = 0.0078; Figure 2J). Furthermore, we performed live staining of LatB-or vehicle-treated hippocampal cultures with antibodies directed against the extracellular epitopes of GluN2B. Increased exposure of GluN2B-containing receptors at the synaptic surface, identified by the presynaptic marker bassoon, was detected upon LatB treatment (Figures 2K,L; synaptic surface GluN2B: Vehicle = 1 ± 0.074 *n* = 13 fields; LatB = 1.531 ± 0.073 *n* = 13 fields in at least three independent experiments. Mann-Whitney Rank Sum Test, *P* ≤ 0.0001), suggesting that the integrity of F-actin is required to set the proper NMDAR composition at the synapse. The efficacy of LatB (300 nM from 10 DIV to 14 DIV) in inducing F-actin depletion in neuronal processes was assessed by Phalloidin staining (Supplementary Figure S2). These data indicate that actin depolymerization, similarly to Eps8 genetic depletion, results in enhanced exposure of GluN2B-containing receptors at the synaptic surface.

**Figure 3:**
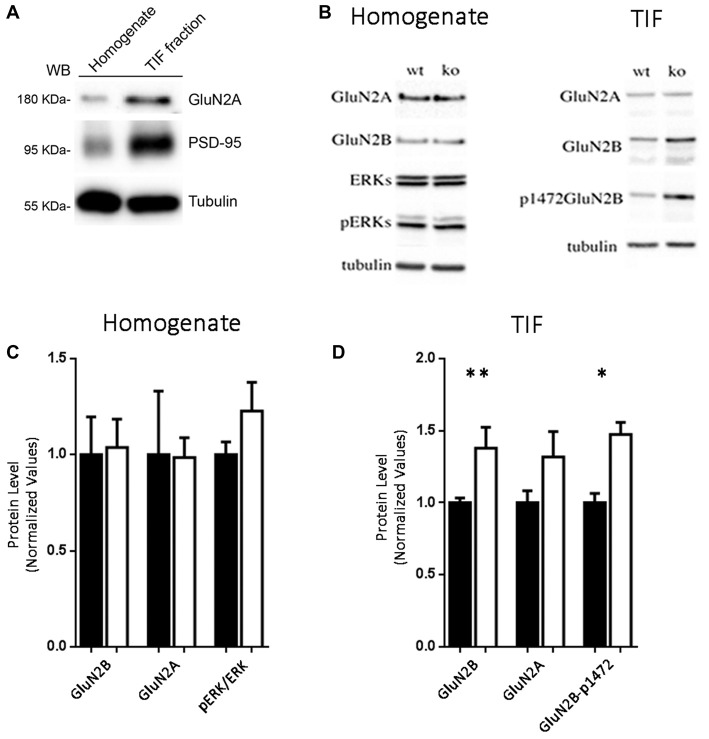
Increased levels of GluN2B-containing and Y1472-GluN2B phosphorylated NMDARs in synaptic triton insoluble fraction (TIF) from Eps8 KO tissue. **(A)** Western blotting (WB) analysis confirmed that TIF preparation was actually enriched in postsynaptic proteins. **(B–D)** WB analysis and relative quantification in homogenate and TIF fractions for the indicated antibodies. All the WB were run twice (data are mean ± SEM, *N* = 4; Kruskal-Wallis test followed by Dunn’s multiple Comparison test *P < 0.05, **P < 0.01).

The role of Eps8 in regulating the NMDA receptor subunit composition at synapses was also examined by Western blotting (WB) analysis in homogenates and postsynaptic TIF (Gardoni et al., 2001a, 2009) prepared from hippocampi of adult WT or Eps8 KO mice (5 months of age). At first, we confirmed that TIF sample was actually enriched in postsynaptic proteins. As shown in Figure 3A, the protein levels of the GluN2A-subunit of NMDAR and of the postsynaptic scaffold protein, PSD-95, were strongly higher in TIF fraction compared to the total homogenate.

No difference in the expression levels of any tested NMDAR subunit was found in the homogenate prepared from hippocampi of Eps8 KO compared to WT adult mice (Figures 3B,C). Conversely, profound differences in NMDAR composition were detected in the postsynaptic compartment as indicated by the immunoblot and relative quantification of TIF fraction (Figures 3B,D). In line with electrophysiological data reported in Figures 2A–G, Eps8 KO tissue was characterized by a significantly higher GluN2B content in the synaptic TIF fraction (Kruskal-Wallis test followed by Dunn’s Multiple Comparison test **P < 0.01, *n* = 4) in the absence of any significant alteration in the GluN2A subunit (Figures 3B,D).

Previous studies reported that direct phosphorylation of the GluN2B subunit by tyrosine kinases in a specific phospho-site is crucial for the dynamic regulation of GluN2B trafficking/turnover (Dunah and Standaert, 2001). Of relevance, GluN2B phosphorylation is mainly restricted to a synaptic fraction (Dunah et al., 2000; Gardoni et al., 2006). We therefore monitored GluN2B Tyr phosphorylation using phospho-specific antibodies raised against the Tyr1472 phospho-site in the C-terminal domain of the receptor subunit (Y1472-GluN2B site). Interestingly, the staining pattern produced in TIF fractions by the GluN2B phospho-specific antibody paralleled the altered level of total GluN2B (Figures 3B–D), thus indicating a significant increase of the GluN2B phosphorylated form in Eps8 KO hippocampal neurons compared to WT (Kruskal-Wallis test followed by Dunn’s Multiple Comparison test *P < 0.05 4, *n* = 4). Furthermore GluN2B Y1472 phosphorylation disrupts the binding to the AP-2 clathrin-associated adaptor protein complex, which targets proteins for endocytosis (Lavezzari et al., 2003). Therefore, an increase in Y1472 GluN2B phosphorylation levels may be linked to decreased GluN2B endocytosis, thus representing a possible mechanism underlying the increase of GluN2B-containing NMDARs in Eps8 KO synapses.

**Figure 4:**
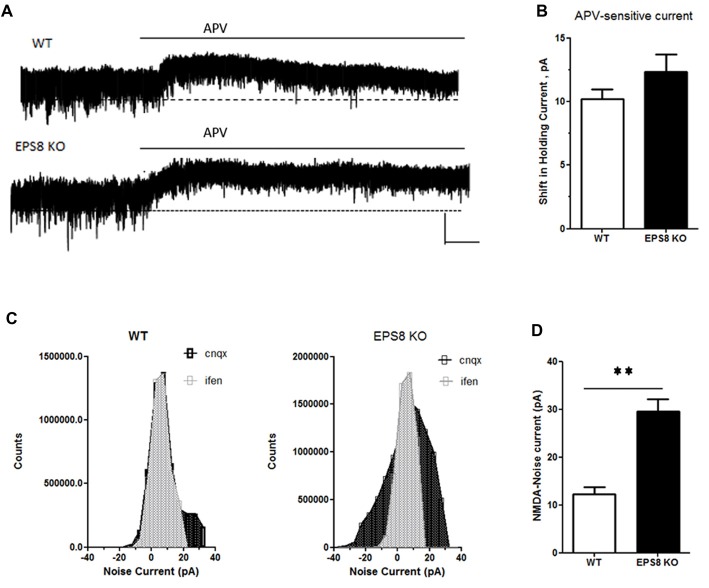
Eps8 KO neurons display an increased NMDA-mediated tonic inward noisy current. **(A)** Examples of NMDA-only mEPSCs/“noise” recorded in [0 Mg^2+^]e ACSF which is abolished by perfusion with the NMDAR antagonist, APV. Dashed lines indicate extrapolated pre-APV shift in holding current. **(B)** Summary of the shift of holding current as a result of 50 μM APV application. **(C,D)** AP-5-associated change in a baseline noise. Quantification of background noise was obtained plotting all values of recordings traces and comparing the fluctuation of values distribution. Data are mean ± SEM (Mann Whitney test, **P < 0.01).

To assess whether the increase of GluN2B-containing receptors is restricted only at synaptic regions, we evaluated the levels of phosphorylated ERK1/2 in hippocampi of mutant adult mice relative to WT. Indeed, it has been shown that synaptic NMDARs promote ERK phosphorylation, whereas extrasynaptic NMDARs lead to ERK dephosphorylation and subsequent inactivation (Hardingham and Bading, 2010). As shown in Figures 3B,C, no increase of phosphorylated ERK1/2 levels (Kruskal-Wallis test followed by Dunn’s Multiple Comparison test, *n* = 4) occurred in Eps8 KO homogenate. This result suggests that the ratio of synaptic vs. extrasynaptic GluN2B-containing NMDARs is not changed probably due to an increase GluN2B-containing NMDARs also at the extrasynaptic level. To further investigate this issue we measured the tonic inward noisy current in [0 Mg^2+^]e and 20 μM CNQX, which has been demonstrated to involve extrasynaptic NMDARs activation in cortical pyramidal neurons (LoTurco et al., 1990; Gottesman and Miller, 2003; Povysheva and Johnson, 2012), possibly due to ambient glutamate or glutamate spillover (Sah et al., 1989; Cavelier et al., 2005; Le Meur et al., 2007). Recordings of NMDA-mediated mEPSCs in [0 Mg^2+^]e at −70 mV from EPS8 KO primary hippocampal neurons displayed a higher noisy signal than WT (Figures 4A,B). Bath application of the NMDAR blocker, APV (50 μM), to WT and EPS8 KO primary cultures reduced noise and abolished the tonic inward current (Figures 4A,B). Moreover, Eps8 KO hippocampal neurons display enhanced background noise respect to WT, measured as the variance of the change of baseline noise at −70 mV before and after bath application of ifenprodil (3 μM; Unpaired *t*-test **P = 0.0044; Figures 4C,D). These data suggest that Eps8 KO neurons display increased content of GluN2B-containing receptors at both synaptic and extrasynaptic sites.

All together these data demonstrate that Eps8 KO hippocampal neurons display altered expression of GluN2A- and GluN2B-containing NMDARs and indicate that mutant neurons show a significantly higher amount of both synaptic and extrasynaptic GluN2B-containing NMDARs.

## Discussion

At the synaptic level, the process of actin remodeling is tightly regulated by a plethora of actin regulating proteins (Sekino et al., 2007; Hotulainen and Hoogenraad, 2010; Bertling and Hotulainen, 2017); among them the actin capping protein, Eps8, is a key factor for dendritic spine morphogenesis (Menna et al., 2013; Stamatakou et al., 2013), and interfering with the capping activity of Eps8 or the genetic elimination of Eps8—such as in Eps8 null neurons—prevents the ability of neurons to undergo NMDA-dependent long term potentiation (Menna et al., 2013). Besides controlling F-actin, Eps8 has been also shown to be part of the NMDAR complex (Offenhauser et al., 2006). In this study, we demonstrate that the lack of Eps8 results in altered subunit composition of synaptic NMDARs, with higher synaptic levels of functional GluN2B- and lower amount of GluN2A-containing receptors. These results indicate that Eps8 is required not only for the process of spine morphogenesis but also for the proper functional maturation of excitatory synapses. The possibility that an abnormal regulation of actin dynamics might be at the origin of the altered maturation of synaptic NMDAR complex in Eps8 KO primary hippocampal neurons is suggested by our results indicating that chronic treatment with LatB results in a similar enhancement in the surface exposure of GluN2B subunits. Consistently, perturbing actin polymerization was previously shown to result in altered NMDA-mediated currents in neurons (Rosenmund and Westbrook, 1993). Previous study have reported that mice overexpressing GluN2B subunit exhibit enhanced hippocampal LTP, prolonged NMDAR currents, and improved memory (Tang et al., 1999) which is maintained also during aging (Cao et al., 2007) suggesting that increasing GluN2B subunit might be beneficial for learning and memory processes. However we previously showed that Eps8 KO neurons are not able to undergo LTP *in vitro* and are characterized by cognitive defects *in vivo* (Menna et al., 2013) that are primarily due to the loss of the capping activity of the protein as they were fully recapitulated *in vitro* by acute inhibition of Eps8 capping activity in WT neurons by using a specific blocking peptide (Menna et al., 2013).

In line with the interaction of Eps8 with NMDAR subunits (Offenhauser et al., 2006), our data indicate a specific defect in NMDAR function in mutant neurons. Although we cannot exclude that additional glutamatergic components may be affected by Eps8 lack, no major defects in AMPA-type glutamate receptor trafficking and composition were detected in Eps8 KO neurons. Indeed, Eps8 KO hippocampal cultures, similarly to wt, were able to undergo homeostatic plasticity in response to neuronal network silencing, a form of Hebbian plasticity requiring AMPAR insertion at the synapse, in a NMDA-independent manner (Wierenga et al., 2006; Turrigiano, 2008; see also Supplementary Figures S1A,B). Also, no changes were detected in the rectification index of Eps8 KO cultures, a value indicative of the presence of different AMPAR subunit subtypes at postsynaptic sites (Hollmann et al., 1991; Derkach et al., 2007; see Supplementary Figures S1C,D).

Numerous studies have demonstrated that the maturation of brain circuitries is usually coincident with the NMDAR subunit switch (e.g., GluN2B-to-GluN2A and GluN3A-to-GluN3B) that occurs at the onset of the critical period of development (Williams et al., 1993; Monyer et al., 1994; Sheng et al., 1994; Paoletti and Neyton, 2007). The NMDAR subunit shift, which therefore marks the transition from immature to mature neural processing (Dumas, 2005), makes the NMDARs extremely vulnerable to genetic and environmental risk factors (Spear, 2000). Of note, the regulation of GluN2A\GluN2B expression and subunit assembly into functional NMDARs is altered in a number of neurological disorders that are characterized also by cognitive defects.

For example, NMDAR hypofunction has been reported in mouse models of schizophrenia (Mohn et al., 1999), increased GluN2B surface expression is observed in Huntington’s disease (Fan and Raymond, 2007; Milnerwood et al., 2010) and genetic evidence clearly implicates association of autism with the NMDA receptor subunit GluN2B (Tarabeux et al., 2011; Yoo et al., 2012). Interestingly, our data show that Eps8 KO adult mice are characterized by an increase in the membrane levels of GluN2B-containing NMDARs (both at the synaptic and extrasynaptic sites), in absence of significant changes in total expression. Notably, this event is mediated by an increase in GluN2B Y1472 phosphorylation that decreases the GluN2B subunit endocytosis.

From our data the possibility therefore emerges that Eps8 is a central element in the proper maturation of postsynaptic excitatory compartment, since the developmental switch from GluN2B-type to GluN2A-type NMDARs is absent or disturbed in Eps8 KO mice. By controlling the actin cytoskeleton, Eps8 orchestrates the structural spine platform (Menna et al., 2013), and the NMDARs function (Offenhauser et al., 2006), and this study), thus linking morphological and functional changes of dendritic spines during development or plasticity phenomena, both in physiological and pathological conditions.

## Author Contributions

RM and EMenna: conceptualization, investigation, methodology, validation, formal analysis and data curation. RM, EMenna and MM: writing—original draft and visualization. RM, SF, FP, SZ, EMarcello, FG and FA: investigation, methodology, data curation and formal analysis. RM, SF, FP, SZ, SP, EMarcello, FG and FA: methodology and data curation. RM, SF and FP: methodology and formal analysis. MM and EMenna: writing—review and editing and conceptualization, investigation, formal analysis, supervision and funding acquisition.

## Conflict of Interest Statement

The authors declare that the research was conducted in the absence of any commercial or financial relationships that could be construed as a potential conflict of interest. The reviewer DPS and handling editor declared their shared affiliation at time of review.

## Footnotes

^1^http://www.informatics.jax.org/allele/MGI:3693626

## Supplementary Material

FIGURE S1 **(A)** Examples of whole-cell patch clamp recordings of mEPSCs from WT and KO hippocampal neurons treated or not with TTX (1 μM) for 48 h to induce homeostatic plasticity. **(B)** Quantitation of mEPSC amplitude in control and TTX conditions showing that both WT and KO neurons were able to undergo homeostatic potentiation (WT ctrl, pA: 22.25 ± 0.7 *n* = 8 cells, WT TTX: 35.45 ± 5 *n* = 10 cells; Mann Whitney test **P* = 0.022; KO ctrl: 22.96 ± 1.9 *n* = 12 cells, KO TTX: 30.4 ± 2.8 14 cells; Unpaired *t*-test, **P* = 0.044). **(C)** Example of AMPA-mediated EPSCs recorded at +40 mV and at −60 mV in WT (top) and EPS8 KO neurons (bottom). **(D)** Summary bar graphs of rectification index analysis (RI: amplitude response at 40 mV/amplitude response at −60 mV) showing no difference in RI value between the two genotypes. All data shown represent the mean ± SEM (Mann Whitney test *P* = 0.5501).

FIGURE S2 Representative images of primary hippocampal neurons treated or not with LatrunculinB (300 nM from 10 DIV to 14 DIV). At 14 DIV neurons were fixed and stained for the postsynaptic markers PSD-95 (green) and for actin filaments by using the specific compound phalloidin. Scale bar depicts 10 μm.

## References

[B1] Allison D. W. Gelfand V. I. Spector I. Craig A. M. (1998). Role of actin in anchoring postsynaptic receptors in cultured hippocampal neurons: differential attachment of NMDA versus AMPA receptors. J. Neurosci. 18, 2423–2436. 10.1523/JNEUROSCI.18-07-02423.1998 9502803 PMC6793094

[B2] Barth A. L. Malenka R. C. (2001). NMDAR EPSC kinetics do not regulate the critical period for LTP at thalamocortical synapses. Nat. Neurosci. 4, 235–236. 10.1038/85070 11224537

[B3] Bedogni F. Cobolli Gigli C. Pozzi D. Rossi R. L. Scaramuzza L. Rossetti G.. (2016). Defects during *Mecp2* null embryonic cortex development precede the onset of overt neurological symptoms. Cereb. Cortex 26, 2517–2529. 10.1093/cercor/bhv078 25979088

[B4] Bertling E. Hotulainen P. (2017). New waves in dendritic spine actin cytoskeleton: from branches and bundles to rings, from actin binding proteins to post-translational modifications. Mol. Cell. Neurosci. 84, 77–84. 10.1016/j.mcn.2017.05.002 28479292

[B5] Cao X. Cui Z. Feng R. Tang Y. P. Qin Z. Mei B.. (2007). Maintenance of superior learning and memory function in NR2B transgenic mice during ageing. Eur. J. Neurosci. 25, 1815–1822. 10.1111/j.1460-9568.2007.05431.x 17432968

[B6] Cavelier P. Hamann M. Rossi D. Mobbs P. Attwell D. (2005). Tonic excitation and inhibition of neurons: ambient transmitter sources and computational consequences. Prog. Biophys. Mol. Biol. 87, 3–16. 10.1016/j.pbiomolbio.2004.06.001 15471587 PMC8906495

[B7] Charrier C. Ehrensperger M. V. Dahan M. Levi S. Triller A. (2006). Cytoskeleton regulation of glycine receptor number at synapses and diffusion in the plasma membrane. J. Neurosci. 26, 8502–8511. 10.1523/JNEUROSCI.1758-06.2006 16914675 PMC6674337

[B8] Cingolani L. A. Goda Y. (2008). Actin in action: the interplay between the actin cytoskeleton and synaptic efficacy. Nat. Rev. Neurosci. 9, 344–356. 10.1038/nrn2373 18425089

[B9] Cohen R. S. Chung S. K. Pfaff D. W. (1985). Immunocytochemical localization of actin in dendritic spines of the cerebral cortex using colloidal gold as a probe. Cell. Mol. Neurobiol. 5, 271–284. 10.1007/bf00711012 4064076 PMC11572803

[B11] Cull-Candy S. G. Brickley S. Farrant M. (2001). NMDA receptor subunits: diversity, development and disease. Curr. Opin. Neurobiol. 11, 327–335. 10.1016/s0959-4388(00)00215-4 11399431

[B10] Cull-Candy S. G. Leszkiewicz D. N. (2004). Role of distinct NMDA receptor subtypes at central synapses. Sci. STKE 2004:re16. 10.1126/stke.2552004re16 15494561

[B12] Derkach V. A. Oh M. C. Guire E. S. Soderling T. R. (2007). Regulatory mechanisms of AMPA receptors in synaptic plasticity. Nat. Rev. Neurosci. 8, 101–113. 10.1038/nrn2055 17237803

[B13] Disanza A. Carlier M. F. Stradal T. E. Didry D. Frittoli E. Confalonieri S.. (2004). Eps8 controls actin-based motility by capping the barbed ends of actin filaments. Nat. Cell Biol. 6, 1180–1188. 10.1038/ncb1199 15558031

[B14] Disanza A. Mantoani S. Hertzog M. Gerboth S. Frittoli E. Steffen A.. (2006). Regulation of cell shape by Cdc42 is mediated by the synergic actin-bundling activity of the Eps8-IRSp53 complex. Nat. Cell Biol. 8, 1337–1347. 10.1038/ncb1502 17115031

[B15] Dumas T. C. (2005). Developmental regulation of cognitive abilities: modified composition of a molecular switch turns on associative learning. Prog. Neurobiol. 76, 189–211. 10.1016/j.pneurobio.2005.08.002 16181726

[B16] Dumoulin A. Triller A. Kneussel M. (2009). Cellular transport and membrane dynamics of the glycine receptor. Front. Mol. Neurosci. 2:28. 10.3389/neuro.02.028.2009 20161805 PMC2820378

[B17] Dunah A. W. Standaert D. G. (2001). Dopamine D1 receptor-dependent trafficking of striatal NMDA glutamate receptors to the postsynaptic membrane. J. Neurosci. 21, 5546–5558. 10.1523/JNEUROSCI.21-15-05546.2001 11466426 PMC6762635

[B18] Dunah A. W. Wang Y. Yasuda R. P. Kameyama K. Huganir R. L. Wolfe B. B.. (2000). Alterations in subunit expression, composition, and phosphorylation of striatal N-methyl-D-aspartate glutamate receptors in a rat 6-hydroxydopamine model of Parkinson’s disease. Mol. Pharmacol. 57, 342–352. 10648644

[B19] Edwards M. Zwolak A. Schafer D. A. Sept D. Dominguez R. Cooper J. A. (2014). Capping protein regulators fine-tune actin assembly dynamics. Nat. Rev. Mol. Cell Biol. 15, 677–689. 10.1038/nrm3869 25207437 PMC4271544

[B20] Erreger K. Dravid S. M. Banke T. G. Wyllie D. J. Traynelis S. F. (2005). Subunit-specific gating controls rat NR1/NR2A and NR1/NR2B NMDA channel kinetics and synaptic signalling profiles. J. Physiol. 563, 345–358. 10.1113/jphysiol.2004.080028 15649985 PMC1665591

[B21] Fan M. M. Raymond L. A. (2007). N-methyl-D-aspartate (NMDA) receptor function and excitotoxicity in Huntington’s disease. Prog. Neurobiol. 81, 272–293. 10.1016/j.pneurobio.2006.11.003 17188796

[B22] Fifková E. Delay R. J. (1982). Cytoplasmic actin in neuronal processes as a possible mediator of synaptic plasticity. J. Cell Biol. 95, 345–350. 10.1083/jcb.95.1.345 6890558 PMC2112353

[B23] Fischer M. Kaech S. Knutti D. Matus A. (1998). Rapid actin-based plasticity in dendritic spines. Neuron 20, 847–854. 10.1016/s0896-6273(00)80467-5 9620690

[B25] Gardoni F. Bellone C. Cattabeni F. Di Luca M. (2001a). Protein kinase C activation modulates α-calmodulin kinase II binding to NR2A subunit of N-methyl-D-aspartate receptor complex. J. Biol. Chem. 276, 7609–7613. 10.1074/jbc.M009922200 11104776

[B27] Gardoni F. Schrama L. H. Kamal A. Gispen W. H. Cattabeni F. Di Luca M. (2001b). Hippocampal synaptic plasticity involves competition between Ca^2+^/calmodulin-dependent protein kinase II and postsynaptic density 95 for binding to the NR2A subunit of the NMDA receptor. J. Neurosci. 21, 1501–1509. 10.1523/JNEUROSCI.21-05-01501.2001 11222640 PMC6762931

[B24] Gardoni F. Picconi B. Ghiglieri V. Polli F. Bagetta V. Bernardi G.. (2006). A critical interaction between NR2B and MAGUK in L-DOPA induced dyskinesia. J. Neurosci. 26, 2914–2922. 10.1523/JNEUROSCI.5326-05.2006 16540568 PMC6673976

[B26] Gardoni F. Mauceri D. Malinverno M. Polli F. Costa C. Tozzi A.. (2009). Decreased NR2B subunit synaptic levels cause impaired long-term potentiation but not long-term depression. J. Neurosci. 29, 669–677. 10.1523/JNEUROSCI.3921-08.2009 19158293 PMC6665154

[B28] Gottesman J. Miller R. F. (2003). N-methyl-D-aspartate receptors contribute to the baseline noise of retinal ganglion cells. Vis. Neurosci. 20, 329–333. 10.1017/s0952523803203114 14570254

[B29] Hanley J. G. (2014). Actin-dependent mechanisms in AMPA receptor trafficking. Front. Cell. Neurosci. 8:381. 10.3389/fncel.2014.00381 25429259 PMC4228833

[B30] Hardingham G. E. Bading H. (2010). Synaptic versus extrasynaptic NMDA receptor signalling: implications for neurodegenerative disorders. Nat. Rev. Neurosci. 11, 682–696. 10.1038/nrn2911 20842175 PMC2948541

[B31] Hollmann M. Hartley M. Heinemann S. (1991). Ca^2+^ permeability of KA-AMPA—gated glutamate receptor channels depends on subunit composition. Science 252, 851–853. 10.1126/science.1709304 1709304

[B32] Hotulainen P. Hoogenraad C. C. (2010). Actin in dendritic spines: connecting dynamics to function. J. Cell Biol. 189, 619–629. 10.1083/jcb.201003008 20457765 PMC2872912

[B100] Joshi P. Gabrielli M. Ponzoni L. Pelucchi S. Stravalaci M. Beeg M.. (2017). Fingolimod Limits Acute Abeta Neurotoxicity and Promotes Synaptic Versus Extrasynaptic NMDA Receptor Functionality in Hippocampal Neurons. Sci. Rep. 7:41734. 10.1038/srep41734 28134307 PMC5278353

[B33] Konietzny A. Bär J. Mikhaylova M. (2017). Dendritic actin cytoskeleton: structure, functions, and regulations. Front. Cell. Neurosci. 11:147. 10.3389/fncel.2017.00147 28572759 PMC5435805

[B34] Lavezzari G. McCallum J. Lee R. Roche K. W. (2003). Differential binding of the AP-2 adaptor complex and PSD-95 to the C-terminus of the NMDA receptor subunit NR2B regulates surface expression. Neuropharmacology 45, 729–737. 10.1016/s0028-3908(03)00308-3 14529712

[B35] Le Meur K. Galante M. Angulo M. C. Audinat E. (2007). Tonic activation of NMDA receptors by ambient glutamate of non-synaptic origin in the rat hippocampus. J. Physiol. 580, 373–383. 10.1113/jphysiol.2006.123570 17185337 PMC2075557

[B36] LoTurco J. J. Mody I. Kriegstein A. R. (1990). Differential activation of glutamate receptors by spontaneously released transmitter in slices of neocortex. Neurosci. Lett. 114, 265–271. 10.1016/0304-3940(90)90574-s 2169598

[B37] Matus A. Bernhardt R. Hugh-Jones T. (1981). High molecular weight microtubule-associated proteins are preferentially associated with dendritic microtubules in brain. Proc. Natl. Acad. Sci. U S A 78, 3010–3014. 10.1073/pnas.78.5.3010 7019915 PMC319489

[B38] Mellone M. Stanic J. Hernandez L. F. Iglesias E. Zianni E. Longhi A.. (2015). NMDA receptor GluN2A/GluN2B subunit ratio as synaptic trait of levodopa-induced dyskinesias: from experimental models to patients. Front. Cell. Neurosci. 9:245. 10.3389/fncel.2015.00245 26217176 PMC4491616

[B39] Menna E. Disanza A. Cagnoli C. Schenk U. Gelsomino G. Frittoli E.. (2009). Eps8 regulates axonal filopodia in hippocampal neurons in response to brain-derived neurotrophic factor (BDNF). PLoS Biol. 7:e1000138. 10.1371/journal.pbio.1000138 19564905 PMC2696597

[B40] Menna E. Zambetti S. Morini R. Donzelli A. Disanza A. Calvigioni D.. (2013). Eps8 controls dendritic spine density and synaptic plasticity through its actin-capping activity. EMBO J. 32, 1730–1744. 10.1038/emboj.2013.107 23685357 PMC3680733

[B41] Milnerwood A. J. Gladding C. M. Pouladi M. A. Kaufman A. M. Hines R. M. Boyd J. D.. (2010). Early increase in extrasynaptic NMDA receptor signaling and expression contributes to phenotype onset in Huntington’s disease mice. Neuron 65, 178–190. 10.1016/j.neuron.2010.01.008 20152125

[B42] Mohn A. R. Gainetdinov R. R. Caron M. G. Koller B. H. (1999). Mice with reduced NMDA receptor expression display behaviors related to schizophrenia. Cell 98, 427–436. 10.1016/s0092-8674(00)81972-8 10481908

[B43] Monyer H. Burnashev N. Laurie D. J. Sakmann B. Seeburg P. H. (1994). Developmental and regional expression in the rat brain and functional properties of four NMDA receptors. Neuron 12, 529–540. 10.1016/0896-6273(94)90210-0 7512349

[B44] Nicholson-Dykstra S. Higgs H. N. Harris E. S. (2005). Actin dynamics: growth from dendritic branches. Curr. Biol. 15, R346–R357. 10.1016/j.cub.2005.04.029 15886095

[B45] Offenhauser N. Castelletti D. Mapelli L. Soppo B. E. Regondi M. C. Rossi P.. (2006). Increased ethanol resistance and consumption in Eps8 knockout mice correlates with altered actin dynamics. Cell 127, 213–226. 10.1016/j.cell.2006.09.011 17018287

[B46] Pak C. W. Flynn K. C. Bamburg J. R. (2008). Actin-binding proteins take the reins in growth cones. Nat. Rev. Neurosci. 9, 136–147. 10.1038/nrn2236 18209731

[B49] Paoletti P. Ascher P. Neyton J. (1997). High-affinity zinc inhibition of NMDA NR1-NR2A receptors. J. Neurosci. 17, 5711–5725. 10.1523/JNEUROSCI.17-15-05711.1997 9221770 PMC6573217

[B48] Paoletti P. Bellone C. Zhou Q. (2013). NMDA receptor subunit diversity: impact on receptor properties, synaptic plasticity and disease. Nat. Rev. Neurosci. 14, 383–400. 10.1038/nrn3504 23686171

[B47] Paoletti P. Neyton J. (2007). NMDA receptor subunits: function and pharmacology. Curr. Opin. Pharmacol. 7, 39–47. 10.1016/j.coph.2006.08.011 17088105

[B50] Povysheva N. V. Johnson J. W. (2012). Tonic NMDA receptor-mediated current in prefrontal cortical pyramidal cells and fast-spiking interneurons. J. Neurophysiol. 107, 2232–2243. 10.1152/jn.01017.2011 22236713 PMC3331604

[B51] Qualmann B. Boeckers T. M. Jeromin M. Gundelfinger E. D. Kessels M. M. (2004). Linkage of the actin cytoskeleton to the postsynaptic density via direct interactions of Abp1 with the ProSAP/Shank family. J. Neurosci. 24, 2481–2495. 10.1523/JNEUROSCI.5479-03.2004 15014124 PMC6729500

[B52] Rosenmund C. Westbrook G. L. (1993). Calcium-induced actin depolymerization reduces NMDA channel activity. Neuron 10, 805–814. 10.1016/0896-6273(93)90197-y 7684233

[B53] Sah P. Hestrin S. Nicoll R. A. (1989). Tonic activation of NMDA receptors by ambient glutamate enhances excitability of neurons. Science 246, 815–818. 10.1126/science.2573153 2573153

[B54] Santucci D. M. Raghavachari S. (2008). The effects of NR2 subunit-dependent NMDA receptor kinetics on synaptic transmission and CaMKII activation. PLoS Comput. Biol. 4:e1000208. 10.1371/journal.pcbi.1000208 18974824 PMC2563690

[B56] Sattler R. Xiong Z. Lu W. Y. Hafner M. MacDonald J. F. Tymianski M. (1999). Specific coupling of NMDA receptor activation to nitric oxide neurotoxicity by PSD-95 protein. Science 284, 1845–1848. 10.1126/science.284.5421.1845 10364559

[B55] Sattler R. Xiong Z. Lu W. Y. MacDonald J. F. Tymianski M. (2000). Distinct roles of synaptic and extrasynaptic NMDA receptors in excitotoxicity. J. Neurosci. 20, 22–33. 10.1523/JNEUROSCI.20-01-00022.2000 10627577 PMC6774093

[B57] Scita G. Nordstrom J. Carbone R. Tenca P. Giardina G. Gutkind S.. (1999). EPS8 and E3B1 transduce signals from Ras to Rac. Nature 401, 290–293. 10.1038/45822 10499589

[B58] Sekino Y. Kojima N. Shirao T. (2007). Role of actin cytoskeleton in dendritic spine morphogenesis. Neurochem. Int. 51, 92–104. 10.1016/j.neuint.2007.04.029 17590478

[B59] Shekhar S. Pernier J. Carlier M. F. (2016). Regulators of actin filament barbed ends at a glance. J. Cell Sci. 129, 1085–1091. 10.1242/jcs.179994 26940918

[B61] Sheng M. Cummings J. Roldan L. A. Jan Y. N. Jan L. Y. (1994). Changing subunit composition of heteromeric NMDA receptors during development of rat cortex. Nature 368, 144–147. 10.1038/368144a0 8139656

[B60] Sheng M. Hoogenraad C. C. (2007). The postsynaptic architecture of excitatory synapses: a more quantitative view. Annu. Rev. Biochem. 76, 823–847. 10.1146/annurev.biochem.76.060805.160029 17243894

[B62] Spear L. P. (2000). The adolescent brain and age-related behavioral manifestations. Neurosci. Biobehav. Rev. 24, 417–463. 10.1016/s0149-7634(00)00014-2 10817843

[B63] Stamatakou E. Marzo A. Gibb A. Salinas P. C. (2013). Activity-dependent spine morphogenesis: a role for the actin-capping protein Eps8. J. Neurosci. 33, 2661–2670. 10.1523/JNEUROSCI.0998-12.2013 23392693 PMC3590009

[B64] Tang Y. P. Shimizu E. Dube G. R. Rampon C. Kerchner G. A. Zhuo M.. (1999). Genetic enhancement of learning and memory in mice. Nature 401, 63–69. 10.1038/43432 10485705

[B65] Tarabeux J. Kebir O. Gauthier J. Hamdan F. F. Xiong L. Piton A.. (2011). Rare mutations in N-methyl-D-aspartate glutamate receptors in autism spectrum disorders and schizophrenia. Transl. Psychiatry 1:e55. 10.1038/tp.2011.52 22833210 PMC3309470

[B66] Tavazzani E. Spaiardi P. Zampini V. Contini D. Manca M. Russo G.. (2016). Distinct roles of Eps8 in the maturation of cochlear and vestibular hair cells. Neuroscience 328, 80–91. 10.1016/j.neuroscience.2016.04.038 27132230

[B67] Tovar K. R. Westbrook G. L. (1999). The incorporation of NMDA receptors with a distinct subunit composition at nascent hippocampal synapses *in vitro*. J. Neurosci. 19, 4180–4188. 10.1523/JNEUROSCI.19-10-04180.1999 10234045 PMC6782704

[B68] Turrigiano G. G. (2008). The self-tuning neuron: synaptic scaling of excitatory synapses. Cell 135, 422–435. 10.1016/j.cell.2008.10.008 18984155 PMC2834419

[B69] Wechsler A. Teichberg V. I. (1998). Brain spectrin binding to the NMDA receptor is regulated by phosphorylation, calcium and calmodulin. EMBO J. 17, 3931–3939. 10.1093/emboj/17.14.3931 9670010 PMC1170728

[B70] Wierenga C. J. Walsh M. F. Turrigiano G. G. (2006). Temporal regulation of the expression locus of homeostatic plasticity. J. Neurophysiol. 96, 2127–2133. 10.1152/jn.00107.2006 16760351

[B71] Williams K. Russell S. L. Shen Y. M. Molinoff P. B. (1993). Developmental switch in the expression of NMDA receptors occurs *in vivo* and *in vitro*. Neuron 10, 267–278. 10.1016/0896-6273(93)90317-k 8439412

[B72] Wyllie D. J. Livesey M. R. Hardingham G. E. (2013). Influence of GluN2 subunit identity on NMDA receptor function. Neuropharmacology 74, 4–17. 10.1016/j.neuropharm.2013.01.016 23376022 PMC3778433

[B73] Wyszynski M. Lin J. Rao A. Nigh E. Beggs A. H. Craig A. M.. (1997). Competitive binding of α-actinin and calmodulin to the NMDA receptor. Nature 385, 439–442. 10.1038/385439a0 9009191

[B74] Yashiro K. Philpot B. D. (2008). Regulation of NMDA receptor subunit expression and its implications for LTD, LTP, and metaplasticity. Neuropharmacology 55, 1081–1094. 10.1016/j.neuropharm.2008.07.046 18755202 PMC2590778

[B75] Yoo H. J. Cho I. H. Park M. Yang S. Y. Kim S. A. (2012). Family based association of GRIN2A and GRIN2B with Korean autism spectrum disorders. Neurosci. Lett. 512, 89–93. 10.1016/j.neulet.2012.01.061 22326929

