# Supplementary figures and images for: Lack of the Actin Capping Protein, Eps8, Affects NMDA-Type Glutamate Receptor Function and Composition
Source: Front Mol Neurosci. 2018 Sep 5;11:313. doi: 10.3389/fnmol.2018.00313 (PMC6133960; doi:10.3389/fnmol.2018.00313)

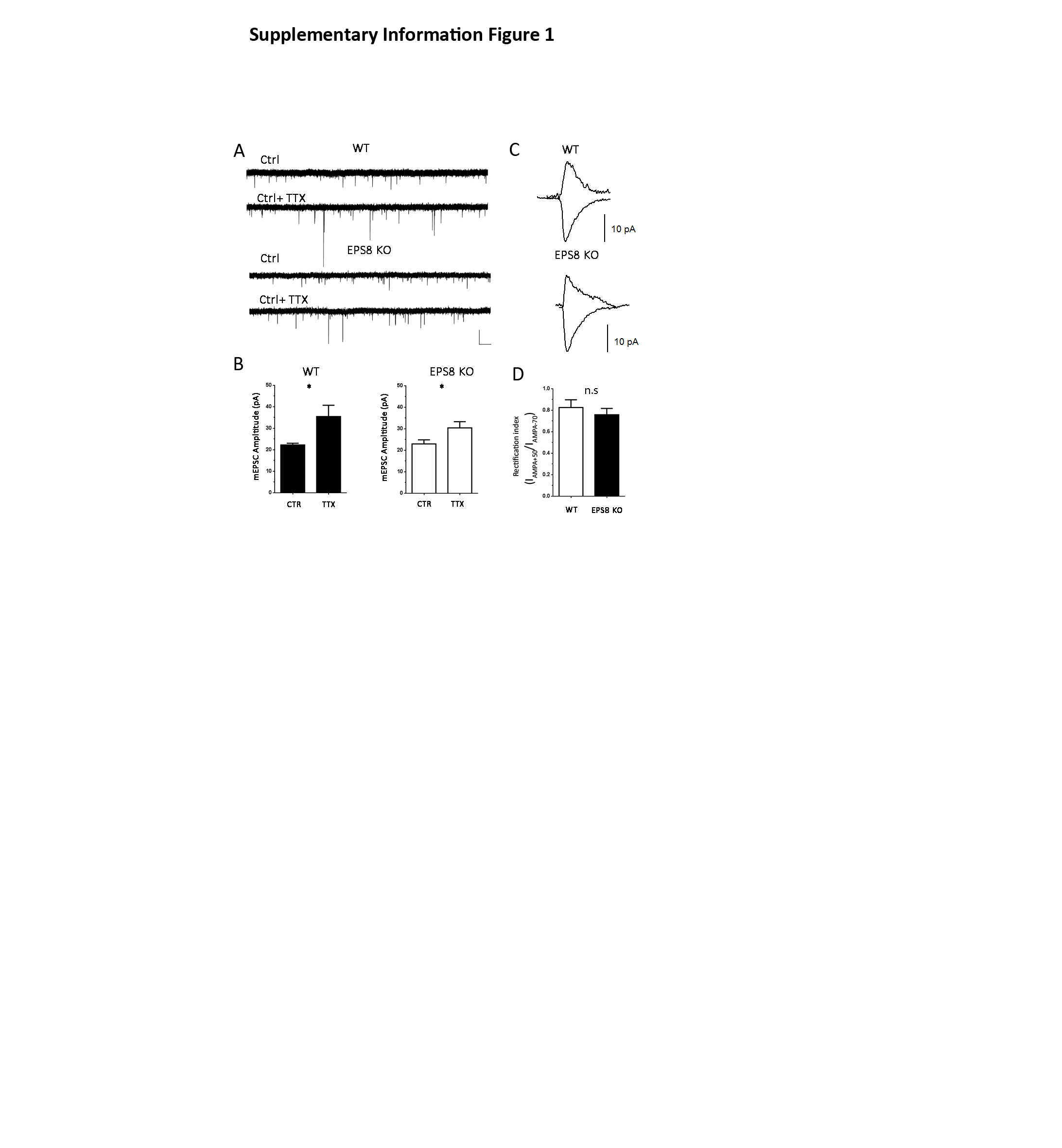

Supplement: FIGURE S1 — (A) Examples of whole-cell patch clamp recordings of mEPSCs from WT and KO hippocampal neurons treated or not with TTX (1 μM) for 48 h to induce homeostatic plasticity. (B) Quantitation of mEPSC amplitude in control and TTX conditions showing that both WT and KO neurons were able to undergo homeostatic potentiation (WT ctrl, pA: 22.25 ± 0.7 n = 8 cells, WT TTX: 35.45 ± 5 n = 10 cells; Mann Whitney test *P = 0.022; KO ctrl: 22.96 ± 1.9 n = 12 cells, KO TTX: 30.4 ± 2.8 14 cells; Unpaired t-test, *P = 0.044). (C) Example of AMPA-mediated EPSCs recorded at +40 mV and at −60 mV in WT (top) and EPS8 KO neurons (bottom). (D) Summary bar graphs of rectification index analysis (RI: amplitude response at 40 mV/amplitude response at −60 mV) showing no difference in RI value between the two genotypes. All data shown represent the mean ± SEM (Mann Whitney test P = 0.5501). [file Image_1.tif]

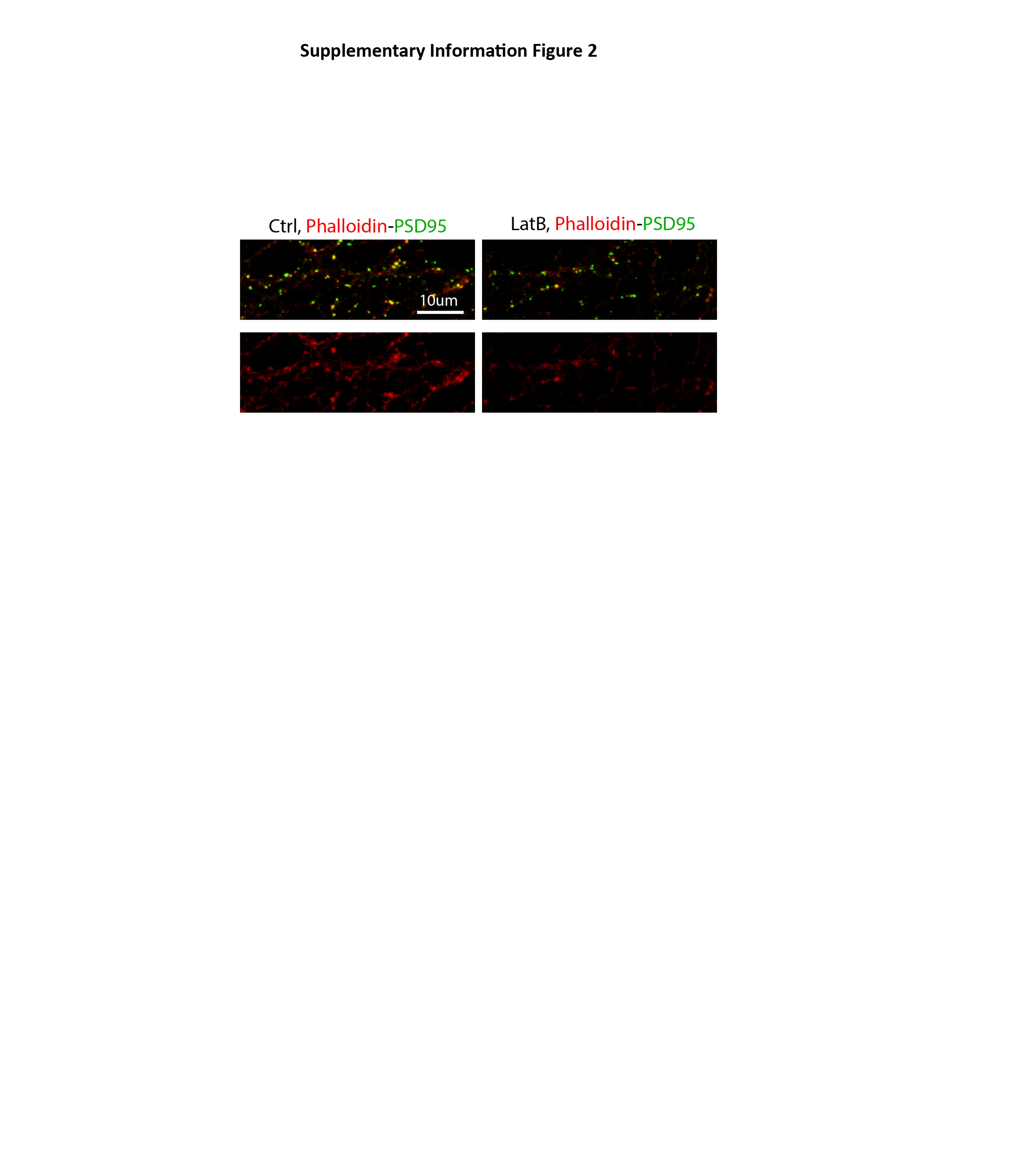

Supplement: FIGURE S2 — Representative images of primary hippocampal neurons treated or not with LatrunculinB (300 nM from 10 DIV to 14 DIV). At 14 DIV neurons were fixed and stained for the postsynaptic markers PSD-95 (green) and for actin filaments by using the specific compound phalloidin. Scale bar depicts 10 μm. [file Image_2.tif]
